# Identification of Novel Single Nucleotide Polymorphisms (SNPs) in Deer (*Odocoileus* spp.) Using the BovineSNP50 BeadChip

**DOI:** 10.1371/journal.pone.0036536

**Published:** 2012-05-08

**Authors:** Gwilym D. Haynes, Emily K. Latch

**Affiliations:** Department of Biological Sciences, Behavioral and Molecular Ecology Research Group, University of Wisconsin – Milwaukee, Milwaukee, Wisconsin, United States of America; Oxford Brookes University, United Kingdom

## Abstract

Single nucleotide polymorphisms (SNPs) are growing in popularity as a genetic marker for investigating evolutionary processes. A panel of SNPs is often developed by comparing large quantities of DNA sequence data across multiple individuals to identify polymorphic sites. For non-model species, this is particularly difficult, as performing the necessary large-scale genomic sequencing often exceeds the resources available for the project. In this study, we trial the Bovine SNP50 BeadChip developed in cattle (*Bos taurus*) for identifying polymorphic SNPs in cervids *Odocoileus hemionus* (mule deer and black-tailed deer) and *O. virginianus* (white-tailed deer) in the Pacific Northwest. We found that 38.7% of loci could be genotyped, of which 5% (n = 1068) were polymorphic. Of these 1068 polymorphic SNPs, a mixture of putatively neutral loci (n = 878) and loci under selection (n = 190) were identified with the F_ST_-outlier method. A range of population genetic analyses were implemented using these SNPs and a panel of 10 microsatellite loci. The three types of deer could readily be distinguished with both the SNP and microsatellite datasets. This study demonstrates that commercially developed SNP chips are a viable means of SNP discovery for non-model organisms, even when used between very distantly related species (the Bovidae and Cervidae families diverged some 25.1−30.1 million years before present).

## Introduction

Single nucleotide polymorphisms (SNPs) are increasingly becoming the marker of choice for investigating contemporary and evolutionary genetic processes (e.g. [1], ). SNPs have many advantages over more traditionally used allozymes, microsatellite loci and chain-termination (Sanger) sequencing of select loci. These include availability in high numbers, presence in coding and non-coding regions, low-scoring error rates, relative ease of calibration between different studies and conformation to simple models of mutation. Furthermore, SNPs can be genotyped using high-throughput protocols that allow thousands of loci to be scored simultaneously, even from low quality DNA samples [2], [5], [6]. In species with fully sequenced genomes (i.e., ‘model’ organisms), panels of SNP markers that cover the entire genome can be devised to allow marker-trait association studies of high statistical power and accuracy (e.g. [7], [8], [9]). SNPs are also useful in researching the genetics of non-model organisms, and can be used in place of or in tandem with microsatellite markers to investigate kinship [10], individual identification [11], parentage inference [12] and population structure [13]. In addition, a SNP panel including both selectively neutral loci and loci under selection could be beneficial in studies of non-model organisms, as neutral loci can be used to make inferences about long-term demographic processes (e.g., migration) whereas loci under selection can be used to differentiate recently diverged lineages or identify genomic regions involved in local adaptation, reproductive isolation or speciation [4], [14], [15].

Developing a panel of SNP markers can be a challenge when working with non-model organisms. While next-generation sequencing technologies have greatly reduced the cost of DNA sequencing [16], performing such sequencing on enough individuals to identify SNPs (with minimal bias) is still outside the resources of many projects. One means of SNP discovery that does not require extensive sequencing is to use commercially available SNP chips developed for a related, well-studied model species. SNP chips are microarrays specifically customized for genotyping known SNP loci, and allow thousands of such loci to be scored simultaneously for two alleles. Recently, SNP chips from agricultural species have been used to identify SNPs in closely related, non-model species. For example, Miller *et al.* [17] identified 868 SNPs in bighorn (*Ovis canadensis*) and thinhorn sheep (*Ovis dalli*) using the OvineSNP50 BeadChip developed for domestic sheep (*Ovis aries*). Similarly, Pertoldi *et al.* [18] used the BovineSNP50 BeadChip developed for cattle (*Bos taurus*) to genotype 2 209 polymorphic loci in European (*Bison bonasus*) and American bison (*B. bison bison* and *B. bison athabascae*). These studies confirm that cross-species application of commercial SNP chips can be a successful strategy for SNP discovery in non-model organisms. This strategy, however, has only been applied to SNP development in non-model species closely related to the focal species. Domestic sheep diverged from bighorn and thinhorn sheep approximately 3.1 million years ago (MYA) [19], while cattle and bison diverged 1.2−2.1 MYA [20]. The use of commercial SNP chips in non-model organisms therefore warrants further investigation regarding their utility in more divergent lineages.

In this study, the potential utility of commercial SNP chip technology for identification of SNPs in non-model organisms is tested between two lineages that diverged approximately 25.1−30.1 MYA, deer (family Cervidae) and cattle (family Bovidae) [21]. The Illumina BovineSNP50 BeadChip developed for commercial SNP genotyping of *B. taurus* is used to genotype DNA samples from a diverse species complex of deer indigenous to North America: mule deer and black-tailed deer (*Odocoileus hemionus* ssp.), and white-tailed deer (*O. virginianus*) [22]. A suite of novel SNPs is characterized, and putatively neutral and selected loci are identified. A range of population genetic analyses are implemented using these SNPs and a panel of 10 microsatellite loci to assess whether the newly identified SNPs behave in a predictable fashion.

## Results

Of the 54 609 SNPs on the chip, 21 131 (38.7%) were scored successfully in at least 90% of individuals, and 1068 of these loci were polymorphic. Minor allele frequency (MAF) is widely used to describe the genetic variability of two-allele SNPs, and refers to frequency of the least common SNP allele. MAF for each locus overall and within each deer lineage is detailed in Table S1. MAF varied across loci and between lineages. The majority of minor alleles were at low frequencies of 0.1 or less, and some loci that were polymorphic overall were monomorphic within a single lineage (Table 1; Table S). To minimize ascertainment bias, all polymorphic SNPs were included in downstream analyses, regardless of the level of genetic variability. The microsatellites were successfully genotyped for 98.6% of alleles, with 4−13 alleles detected at each locus.

**Table 1:** Minor allele frequencies for each deer lineage.

Frequency	All Deer		Mule Deer		Black-Tailed Deer		White-Tailed Deer	
	# loci	%	# loci	%	# loci	%	# loci	%
0*	NA	NA	639	60%	634	59%	599	56%
0.0−0.1	691	64.70%	232	21.72%	200	18.73%	0	0.00%
0.1−0.2	229	21.44%	69	6.46%	71	6.65%	195	18.26%
0.2−0.3	61	5.71%	44	4.12%	73	6.84%	92	8.61%
0.3−0.4	32	3.00%	27	2.53%	35	3.28%	99	9.27%
0.4−0.5	55	5.15%	57	5.34%	55	5.15%	83	7.77%
**Total # of polymorphic loci:**	1068		429		434		469	

* A MAF value of 0 indicates that loci were polymorphic overall but monomorphic within a particular lineage.

The analysis in lositan identified 878 SNP loci as neutral, 116 as being under positive selection and 74 under balancing selection after adjustment for multiple testing (Table S1). Departures from HWE were non-significant in all analyses (Table 2). The standard deviation was high for all genetic diversity measures (Table 2), likely because of the small sample sizes analyzed. Expected heterozygosity (H_E_) and observed heterozygosity (H_O_) were generally lower for SNPs than for microsatellites, though this difference between marker types was only significant in mule deer (Table 2). F_IS_ differed markedly between species and datasets but was also generally lower for SNPs than for microsatellites (Table 2). The overall P_(ID)_ (Table 2) was extremely low for both the 1068 polymorphic SNPs (3.4×10^−162^) and the 878 neutral SNPs (3.0×10^−123^), attesting to the high discriminatory power of these markers. Although P_(ID)_ was an order of magnitude higher for microsatellites (3.6×10^−12^; Table 2 ) than for SNPs, this value still indicates a very high discriminatory power for the microsatellites as it is well above the P_(ID)_ value of at least 10^-3^–10^-4^ recommended for wildlife forensic applications [23].

**Table 2:** Hardy-Weinberg Equilibrium (HWE) *p*-values, expected heterozygosity (H_E_), observed heterozygosity (H_O_), and F_IS_ for mule deer (MD), black-tailed deer (BTD) and white tailed deer (WTD) with associated p values.

	10 microsatellites	1068 polymorphic SNPs	878 neutral SNPs
MD			
HWE	0.4587	0.9912	1.0000
H_E_	0.6358 (0.1384)	0.2389 (0.1619)	0.2259 (0.1566)
H_O_	0.5417 (0.1582)	0.2545 (0.2290)	0.2273 (0.1858)
F_IS_	0.1539 (0.0596)	−0.0683 (0.0169)	−0.0067 (0.0181)
P_(ID)_	1.4×10^−9^	5.7×10^−103^	2.2×10^−85^
BTD			
HWE	0.982	0.9412	1.0000
H_E_	0.5916 (0.2495)	0.2597 (0.1617)	0.2479 (0.1581)
H_O_	0.5659 (0.2618)	0.2538 (0.2122)	0.2278 (0.1749)
F_IS_	0.0454 (0.0576)	0.0236 (0.0165)	0.0842 (0.0173)
P_(ID)_	8.5×10^−11^	1.1×10^−112^	9.1×10^−97^
WTD			
HWE	0.5881	1.0000	1.0000
H_E_	0.5446 (0.2072)	0.4292 (0.1660)	0.4065 (0.1368)
H_O_	0.4375 (0.2588)	0.3966 (0.2795)	0.3568 (0.2509)
F_IS_	0.2222 (0.1408)	0.0875 (0.0258)	0.1406 (0.0305)
			
Overall P_(ID)_	3.6×10^−12^	3.4×10^−162^	3.0×10^−123^

Expected probability of identity, P_(ID)_, is estimated overall for each subset of DNA loci and individually for MD and BTD. P_(ID)_ could not be calculated individually for WTD due to limited sample size.

**Figure 1:**
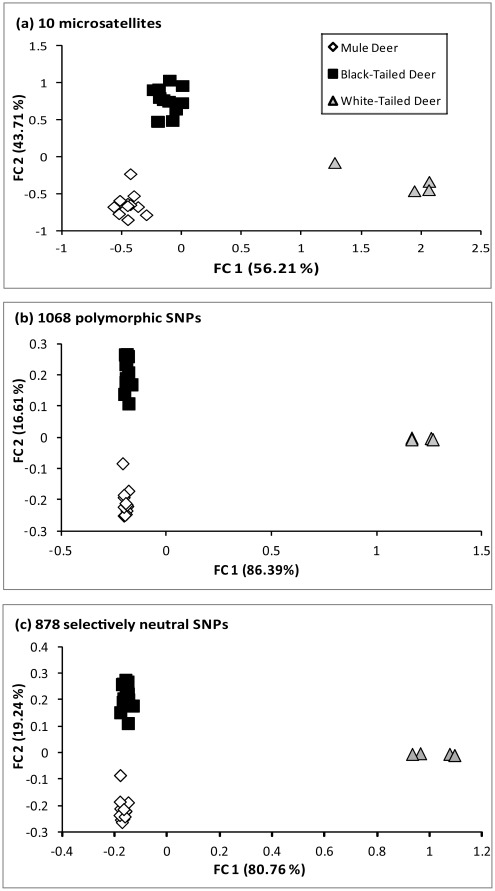
Factorial component analysis (FCA) of mule deer (MD), black-tailed deer (BTD) and white-tailed deer (WTD) estimated using (a) microsatellites, (b) all 1068 polymorphic SNPs, and (c) the 878 SNPs identified as selectively neutral.

All three deer lineages were distinguished from each other in all analyses and for all datasets. Fisher’s exact test in genepop returned significant (*p-*value <0.001) departures from panmixia in all pairwise comparisons. The analyses in structure all returned *K* = 3. For the microsatellites, the highest Δ*K* value was at *K* = 3, with the mule deer, black-tailed deer and white-tailed deer each partitioned into distinct clusters (Table 3). Both SNP datasets initially returned a highest Δ*K* value at *K* = 2, where mule deer and black-tailed deer were clustered together to the exclusion of white-tailed deer. As structure only identifies the upper most level of population structure [24], the analyses were rerun without the white-tailed deer to determine if additional substructure could be identified within the cluster containing mule deer and black-tailed deer. The highest Δ*K* in both these subsequent analyses was two (Table 4), with the mule deer and black-tailed deer partitioned into discrete genetic clusters. Finally, FCA readily separated each of the three lineages into distinct clusters. These clusters were completely discrete for the microsatellites (Figure 1A), while the SNPs placed the mule deer and black-tailed deer into partially overlapping but still discernible clusters (Figure 1B, 1C).

**Table 3:** Analysis in STRUCTURE for all 28 deer using 10 microsatellites, all 1068 polymorphic SNPs and the 878 putatively neutral SNPs.

K	Ln P(D)			ΔK
	Iteration 1	Iteration 2	Iteration 3	
*10 microsatellites*				
1	−892.7	−891.3	−892.3	NA
2	−782.7	−779.5	−780.9	15.2
3	−694.4	−695	−693.9	168.1
4	−700.1	−698.5	−702.6	0.1
5	−708.4	−704.4	−706.9	1.0
6	−709.8	−711.8	−710.8	NA
*1068 SNPs*				
1	−16594.8	−16651.1	−16600.2	NA
2	−11851.4	−11852.3	−11846.1	1491.8
3	−12113.5	−12045.8	−12087.2	9.6
4	−12755.5	−11681	−11523.7	13.6
5	−34076.5	−17164	−11884.7	0.7
6	−41501	−12490.6	−12312.2	NA
*878 SNPs*				
1	−12831.7	−12831.5	−12831.5	NA
2	−10167.3	−10169.8	−10176.2	557.4
3	−9566.1	−10313.7	−10328.4	1.6
4	−12234	−9841.1	−9858.7	0.3
5	−10148.8	−10793.1	−11588.8	0.7
6	−10155.8	−11214.6	−10246.4	NA

**Table 4:** Analysis in STRUCTURE using only mule deer and black-tailed deer for all 1068 polymorphic SNPs and 878 putatively neutral SNPs.

K	Ln P(D)			ΔK
	Iteration 1	Iteration 2	Iteration 3	
*1068 SNPs*				
1	−9278.7	−9300.2	−9281.9	NA
2	−8510.7	−8516.8	−8499.2	96.9
3	−8585.6	−8605.2	−8598.6	9.7
4	−8594.6	−8589.4	−8578.7	4.4
5	−8624	−8625.2	−8593.7	2.6
6	−8598	−8594.7	−8588.8	NA
*878 SNPs*				
1	−7984.2	−7961.8	−7961.6	NA
2	−7286.1	−7289.6	−7294.4	174.0
3	−7334.2	−7336.4	−7336.8	33.9
4	−7331.6	−7342.6	−7328.1	1.1
5	−7337.9	−7343.8	−7339.4	0.5
6	−7341.2	−7331.3	−7362.7	NA

All datasets and all measures of genetic distance clearly identified mule deer and black-tailed deer as more closely related to one another than either was to white-tailed deer (Figure 1). This pattern is consistent with previous studies of morphological characters [25], nuclear DNA [26], [27] and the Y-chromosome [28]; although it should be noted that mitochondrial DNA studies have revealed a different pattern, with mule deer and white-tailed deer being most closely related [26], . F_ST_ was higher for SNPs than for microsatellites in two of the three comparisons (Figure 1), likely because F_ST_ has a tendency to be reduced by high levels of polymorphism [32]–[35]. *D* and *D_m_* were far higher for microsatellites than for SNPs (Figure 1). *D* is an explicit measure of allele frequency differences between sample groups that makes no correction for high numbers of alleles. High mutation rates (and therefore large numbers of alleles) typical of microsatellites therefore lead to higher values of *D* relative to loci with low mutation rates and low numbers of alleles, such as SNPs [36]. *D_m_* is similarly elevated increased by high levels of heterozygosity [37], and is likely elevated here by the higher H_O_ values detected for microsatellites in mule deer and black-tailed deer than for SNPs (Table 2).

## Discussion

Of the 54 609 loci on the BovineSNP50 BeadChip, 21 131 (38.7%) SNPs were successfully genotyped in at least 90% of individuals, and 1068 (2.0% of the total; 5.1% of genotyped loci) were polymorphic in deer. In comparison, Pertoldi et al. [18] successfully genotyped a far greater proportion of loci (96.7–98.7%) and detected 4% of loci as polymorphic using the same SNP chip in bison; and Miller et al. [17] successfully genotyped over 90% of loci in closely related species of sheep using the OvineSNP50 BeadChip, yet found only 1.7% of sites to be polymorphic (868 out of a total of 49 034 loci). The lower rate of genotyping success in this study when compared with Pertoldi et al. [18] and Miller et al. [17] is expected, given the 25.1−30.1 million year divergence between Bovidae (*B. taurus*) and Cervidae (*O. hemionus* and *O. virginianus*) [21]. The level of polymorphism, however, is unexpectedly high and could result from historically high population sizes of mule deer, black-tailed deer and white-tailed deer in North America [24]. In contrast, the bison species analyzed by Pertoldi et al. [18] have undergone several severe population bottlenecks, while the wild sheep species investigated by Miller et al. [17] live in relatively small, isolated populations. The identification of 1068 novel, polymorphic SNPs in this study demonstrates that commercial SNP chip technology is a viable and potentially underutilized means of discovering SNP loci in non-model species, even when used between highly divergent lineages.

Both neutral loci and loci potentially under selection were detected in this study, including 878 neutrally evolving, 116 under the influence of positive selection, and 74 influenced by balancing selection (Table S1). A suite of loci that includes both neutral and selected loci will be useful for a variety of applications. Most population genetic analyses, for example, assume that the genetic markers employed are selectively neutral. Loci under positive selection, however, can be essential in distinguishing between recently diverged species and populations that are otherwise difficult to distinguish using neutral makers [14], [38]. Characterizing genomic regions under balancing selection could identify advantageous genes and alleles that move between populations, such as loci involved in disease resistance (e.g., [39]). Thus, a necessary first step in any genetic study is to accurately characterize suites of loci that match study objectives and ensure the application of appropriate analytical models and correct interpretation of results.

**Figure 2:**
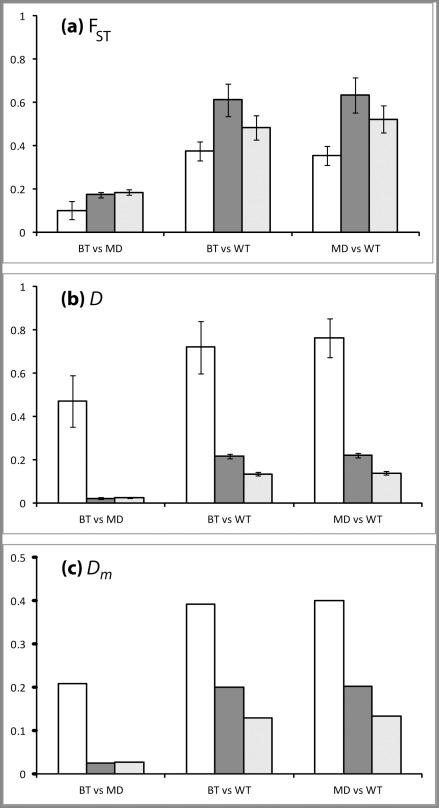
Genetic distance measures estimated between mule deer, black-tailed deer and white-tailed deer using 10 microsatellites (white), all 1068 polymorphic SNPs (dark grey) and 878 putatively neutral loci (pale grey). (a) F_ST_ (with standard deviation), (b) Jost’s *D* (with standard error) and (c) Nei’s minimum distance, *D_m_*.

Population genetic inferences made with the SNPs identified here were consistent with current taxonomic nomenclature and with previous studies of nuclear [27] and Y-chromosome [28] DNA and morphological characters [25] that identified mule and black-tailed deer as closely related and white-tailed deer as a more divergent evolutionary lineage. All measures of genetic distance (F_ST_, *D* and *D_m_*) reported lower differentiation between mule deer and black-tailed deer than between white-tailed deer and either *O. hemionus* lineage (Figure 2). Consistent with the analyses of microsatellites performed here, the three lineages were clearly delineated using exact tests, assignment tests, and FCA using the dataset of all 1068 polymorphic SNPs or the 878 neutral SNPs. Extremely low P_(ID)_ values both overall and within individual lineages suggests that these SNPs would be very useful for fine-scale population genetic analyses requiring unambiguous individual identification. In this study, we used only ‘pure’ representatives of each lineage (as identified by previous genetic analyses; [40]). Further characterization of these SNPs would be necessary to determine their power and accuracy for delineating lineages in areas of sympatry where individuals may be of mixed ancestry.

The level of within-population inbreeding (F_IS_) differed markedly between datasets (Table 2) and warrants further explanation here. The F_IS_ statistic ranges from −1 to 1, with negative values indicating an excess of heterozygosity and positive values indicating excess homozygosity relative to expectations under HWE. For each lineage, deer were sampled from disparate locations, and as such are expected to belong to different populations and to therefore return positive F_IS_ values consistent with homozygote excess (Wahlund effect). In accordance with these expectations, positive F_IS_ values were returned for all lineages for microsatellites (although F_IS_ was not significantly different from zero in white-tailed deer) and for SNPs in black-tailed deer and white-tailed deer. In contrast, statistically significant negative F_IS_ values were returned in mule deer when all 1068 SNPs or the 878 neutral SNPs were analyzed (Table 2). The unexpected heterozygote excess in the SNP data in the mule deer lineage could be caused by a high proportion of low-frequency alleles in mule deer which would in turn lead to an artificially high H_O_. Of the 429 loci that were polymorphic in mule deer, 54% (n = 232) had a minor allele frequency (MAF) less than 0.1 (Table 1). This was higher than the proportion of similarly low-frequency alleles found in black-tailed deer (46%; 200 of 434 polymorphic loci within the black-tailed deer lineage) and white-tailed deer, where the MAF could not be less than 0.125 on account of only 4 individuals being analyzed (if at a given locus only one of the four individuals is heterozygous, the MAF of that locus will be 0.125) (Table 1). Multilocus genotypes from additional individuals would be necessary to more fully evaluate potential mechanisms for the observed heterozygote excess in mule deer.

Any process of SNPs discovery carries some risk of ascertainment bias, where the overall pattern of genetic diversity is not accurately represented by the sampled SNPs. In general, small screening panel size, overly stringent SNP identification algorithms, and bias toward polymorphic loci in SNP selection can lead to inaccurate inferences of genetic diversity, population genetic structure, and phylogenetic relationships [5], . The small sample size of deer initially screened for SNPs in the present study will almost certainly have led to some polymorphic sites not being detected, in particular those sites harboring rare alleles. In addition, the screening of SNPs identified in *B. taurus* for use in *O. hemionus* and *O. virginianus* is likely biased in favor of conserved genomic regions that still retain polymorphisms ancestral to the divergence between Cervidae and Bovidae. Such loci may not be representative of the evolutionary changes that have since occurred within the Cervidae family. The selection of SNPs for the Bovine SNP50 BeadChip that are distributed in a roughly even fashion across the *B. taurus* genome, however, should minimize the effects of this bias. Downstream applications can avoid compounding ascertainment bias by randomly selecting a panel of SNPs for analysis, rather than using only SNPs that exceed a minimum, predefined level of polymorphism [5].

One of the most attractive incentives for using model species to identify SNPs in non-model species is the availability of annotations that link SNP variation to DNA sequences and ultimately to biological processes. Although no deer genomes have yet been fully sequenced and annotated, the genomic location of each SNP identified in this study can be mapped on various versions of the *B. taurus* genome (e.g., the Btau 4.2 assembly, compiled by the Bovine HapMap Consortium, or the UMD3.1 assembly, compiled by the Center for Bioinformatics and Computational Biology at the University of Maryland). The position of each SNP on both Btau4.0 and UMD3.1 is provided in Table S1. However, the level of divergence between our model and non-model species (25–30 MYA) may not permit accurate chromosomal locations to be determined for all identified SNPs. Multiple chromosome rearrangements have occurred in the Bovidae and Cervidae lineages since their divergence, which is especially evident in a change in karyotype from 2n = 70 in cervids *O. virginianus* and *O. hemionus* to 2n = 60 in the bovid *B. taurus* [44]. In spite of these large-scale rearrangements, alignment of deer DNA sequences to the *B. taurus* genome has been successful for next-generation sequences generated from *O. virginianus* [45], presumably owing to regional synteny. Still, caution is warranted when interpreting results obtained from alignments between such divergent lineages.

The SNPs characterized in this study would likely be useful in a variety of applications for an array of cervid species, given the high cross-species amplification success we observed. Neutral SNPs can be readily applied to more traditional population genetic analyses, such as characterizing population structure, quantifying genetic diversity and inferring migration rates. Loci under natural selection could be used to investigate genetic mechanisms underpinning natural selection and adaptation, or to differentiate recently diverged populations, species and ecotypes that are otherwise difficult to distinguish using neutral loci [46]. Such investigations are relevant not only for evolutionary research but also for conservation and management of mule deer, black-tailed deer and white-tailed deer. In addition to being important game species, the U.S. Fish and Wildlife Service lists the Cedros Island mule deer (*O. h. cerrosensis*), Florida Key white-tailed deer (*O. v. calvium*) and Columbian white white-tailed deer in western Oregon (*O. v. leucurus*) as ‘Endangered’ [47]. White-tailed deer are also threatened in Venezuela by overhunting and habitat loss [48]. Thorough delimitation of subpopulation boundaries, identification of locally adapted populations and characterization of genetic diversity patterns will therefore be highly useful in informing regional conservation and management strategies. These commercial SNP chips could even be applied to other cervids of conservation or management concern; for example, those listed as threatened on the IUCN Red List [49] (hog dear, *Axis* spp, revised to genus *Hyelaphus* in [50]; Père David’s deer, *Elaphurus davidianus*; Patagonian huemul, *Hippocamelus bisulcus*).

This study demonstrates the potential utility of commercially available SNP chip technology for identifying SNP loci in non-model organisms. As polymorphic SNPs were identified between lineages that diverged up to 30.1 MYA, SNP chips developed for model organisms can likely identify SNPs in a far wider range of organisms than previously realized. The porcine, ovine, equine and bovine SNP chips, for example, could be used to collectively to develop a panel of SNPs for wide range of highly divergent ungulates; while SNP chips developed for dogs (*Canis lupus familiaris*) could likely identify polymorphic SNPs in a wide range of Carnivora species that would otherwise require extensive DNA sequencing. The cross-species utilization of SNP chips is therefore an exciting avenue of future research.

## Materials and Methods

### Ethics Statement

Samples were collected by Department of Natural Resources staff in Washington and Oregon from hunter-harvested animals between 2003 and 2009. Ethics approval was not required or sought for this research, as the samples were hunter-harvested and thus not collected specifically for this study, and no additional observational or field data were collected.

### Study Organism

Mule deer and black-tailed deer are both classified as *O. hemionus*. Morphological [51], [52] and genetic studies [26], [28], [53], [54], however, strongly support the separation of this species into two highly distinct lineages that diverged in allopatry during the last glacial maximum. Black-tailed deer include subspecies *O. h. columbianus* and *sitkensis* and are found throughout the Pacific Northwest, west of the Cascade Mountains and north to Alaska along the Pacific Coast. Mule deer include subspecies *O. h. hemionus, fulginatus, californicus, inyoensis, eremicus, crooki, peninsulae, sheldoni,* and *cerrosensis*, and are found east of the Cascade Mountains and throughout western and central North America, Canada, and Mexico. White-tailed deer (*O. virginianus*) are more widespread than *O. hemionus*, being found throughout northern South America, Central America, Mexico, central and eastern North America and in a number of isolated populations in western North America. White-tailed deer can be subdivided into as many as 38 subspecies [45], [55], [56]. All three types of deer within this species complex show extensive local adaptation and population structuring [53], [57], [58], yet all have a conserved karyotype of 2n = 70 chromosomes [44] and are capable of extensive hybridization and introgression in regions of sympatry [26], [28], [40], . Notably, all three lineages overlap within our study area in western Oregon, making this region a natural experiment for testing specific hypotheses about such evolutionary processes as hybridization, local adaptation, and reproductive isolation. However, for the purposes of this study, only ‘pure’ samples from each lineage were used (as identified in previous genetic analyses; [40]).

### Sample Collection and DNA Genotyping

**Figure 3:**
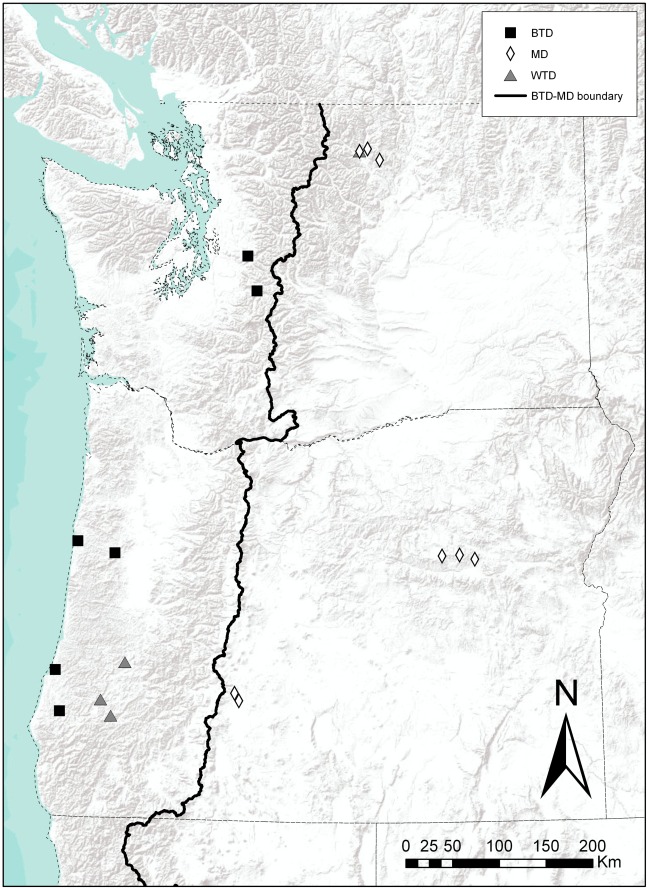
Map of sampling locations for mule deer (MD), black-tailed deer (BTD) and white-tailed deer (WTD).

To evaluate the feasibility of cross-species SNP chip genotyping as a means of SNP discovery, tissue samples were collected from twelve mule deer, twelve black-tailed deer and four white-tailed deer in Washington and Oregon, USA (Figure 3) between 2003 and 2009. Previous genetic analyses identified these deer as ‘pure’ representatives of their respective lineages, i.e., no evidence inter-lineage ancestry [40]. Genomic DNA for each of the 28 deer sampled was genotyped at a commercial lab (Genetic Visions, Inc.) using an Illumina BovineSNP50 Genotyping BeadChip. In addition, 10 selectively neutral microsatellite loci (BM848, Odh_C, Odh_E, Odh_K, C273, Odh_G, Odh_P, Odh_O, RT24, and T40) were PCR-amplified and genotyped according to Latch et al. [67] in all individuals so that statistical inferences made with SNPs could be compared with microsatellites.

### Identification of Neutral Loci and Loci Under Selection

Genetic analyses of wild populations depend on accurately characterizing whether the genetic loci used are under selection. Theoretical models in population genetics typically assume that the markers employed are selectively neutral; including loci under selection can bias inferences about migration rates, genetic diversity, population genetic structure, and phylogenetic relationships. Loci should therefore be screened for signatures of selection prior to population genetic analyses, in order to ensure that appropriate analytical models are used and results are interpreted correctly [15], [43]. Genomic studies, in contrast, are primarily concerned with identifying genes or genomic regions involved in evolutionary processes and can hence benefit from specifically targeting genomic regions suspected to be under selection (e.g. [68]). To identify SNPs potentially under selection in the present study, the F_ST_-outlier method [69] was implemented in the Bayesian program lositan [70]. lositan simulates the expected distribution of Wright’s inbreeding coefficient F_ST_ *vs* expected heterozygosity (H_E_) for a given set of genetic markers under the island model of migration [71]. Loci under positive selection are expected to show greater levels of interpopulation differentiation than neutral loci (i.e., higher F_ST_/H_E_ ratio), whereas loci under balancing selection are expected to show lower levels (i.e., lower F_ST_/H_E_ ratio) of differentiation [72]. lositan was run for 10 000 000 simulations, under the “neutral” mean F_ST_ and forced mean F_ST_ settings, with a two-tailed significance level of 0.05. The mule deer, black-tailed deer, and white tailed deer were designated as different ‘populations’ in the analysis. *P*-values were adjusted for multiple testing using the B-Y method of false discovery rate correction [73] in the R-project package *multtest* [74].

### Statistical Analyses

The statistical properties of the newly identified SNPs were compared with the 10 neutral microsatellite loci to verify that the SNPs were behaving in a predictable fashion. A range of common statistical analyses were implemented using all 1068 polymorphic SNPs identified here (see Results), the 878 SNPs identified as selectively neutral (see Results) and the 10 microsatellite loci to characterize population genetic structure in mule deer, black-tailed deer, and white-tailed deer. Departures of genotype frequencies from expectations under Hardy-Weinberg equilibrium (HWE) were tested using Fisher’s exact test in genepop 4.1 [75]. Heterozygosities were estimated in arlequin 3.5.1.2 [76], and F_IS_ for each deer lineage was calculated in genetix [77]. The unbiased theoretical expected probability of identity P_(ID)_ was calculated for each suite of loci over all deer and within the mule deer and black-tailed deer lineages [23], [78].

To determine if either suite of SNPs (1068 polymorphic loci or 878 neutral loci) could be used to distinguish between mule deer, black-tailed deer and white-tailed deer, significant differences in allele frequencies were assessed using Fisher’s exact test in genepop 4.1 [75]. Assignment tests were also performed in structure 2.3.3 [79], [80] under the Allele Frequencies Correlated Model and Admixture Model. The newly developed Sampling Locations as Priors Model was also used, as this model incorporates pre-defined sample group information (in this case, each individual was identified *a priori* as mule deer, black-tailed deer or white-tailed deer) to allow population structure to be detected at lower levels of divergence and with less data than earlier versions of structure [81]. Assignment tests were run for *K* = 1−6, with 50 000 burn-in steps and 500 000 iterations for each value of *K*. Tests were performed three times for each value of *K*, and the Δ*K* statistic of Evanno et al. [24] was used to determine the most likely value of *K* for each data set. Factorial correspondence analysis (FCA) was implemented in genetix 4.05.2 [77] in order to represent genetic relationships among individual deer graphically.

Three measures of genetic distance were calculated to further confirm that the newly identified SNPs exhibit patterns of genetic variation and structure in accordance with theoretical expectations. Weir and Cockerham’s [82] measure of F_ST_ was calculated in genetix [77], and the standard deviation was estimated using 10 000 permutations. The more recently developed Jost’s *D* [83] was estimated in genodive [84] and the standard error calculated against a background of 10 000 permutations. Nei’s minimum genetic distance, *D_m_* [37], was estimated in populations 1.2.31 [85]. F_ST_ is one of the most commonly used measures of genetic differentiation and is used in lositan to detect loci under selection, despite being strongly affected by high levels of polymorphism [32]–[35]. *D* provides an unbiased quantification of differences in allele frequencies between populations without being affected by levels of genetic diversity and heterozygosity the way F_ST_ and its analogues are [83]. *D_m_* performs well in recently diverged lineages and when mutation rate is low [37], and is therefore well suited for SNP data (low mutation rates and numbers of alleles [86]) and the study system (recently diverged lineages [53]).

## Supporting Information

Table S1 **Genome location, outlier-analysis in LOSITAN and Minor Allele Frequency (MAF) data of the 1068 polymorphic SNPs identified in** ***O. hemionus and O. virginianus*** **.** Only SNPs that were genotyped in at least 90% of individuals were included in the analysis. The chromosomal position of each SNP on the Bos taurus genome assemblies UDM3.0 and BTAU4.0 is included. N/A values in the UMD3.0 assembly indicate that SNPs that are not mapped to this genome assembly. Zero values on the BTAU4.0 assembly are indicative of SNPs that could not be mapped to this assembly.
(XLS)
